# The construction of machine learning-based predictive models for high-quality embryo formation in poor ovarian response patients with progestin-primed ovarian stimulation

**DOI:** 10.1186/s12958-024-01251-5

**Published:** 2024-07-10

**Authors:** Yu-Heng Xiao, Yu-Lin Hu, Xing-Yu Lv, Li-Juan Huang, Li-Hong Geng, Pu Liao, Yu-Bin Ding, Chang-Chun Niu

**Affiliations:** 1 Chongqing Medical University, Chongqing, 400016 China; 2 Department of Laboratory, Chongqing General Hospital, Chongqing, 401121 China; 3 Department of Obstetrics and Gynecology, Women and Children’s Hospital of Chongqing Medical University, Chongqing, 401147 China; 4 The Reproductive Center, Sichuan Jinxin Xinan Women and Children’s Hospital, Chengdu, Sichuan 610011 China; 5 Department of Pharmacology, Academician Workstation, Changsha Medical University, Changsha, 410219 China

**Keywords:** Poor ovarian response, Progestin-primed ovarian stimulation, High-quality embryo, Machine learning

## Abstract

**Objective:**

To explore the optimal models for predicting the formation of high-quality embryos in Poor Ovarian Response (POR) Patients with Progestin-Primed Ovarian Stimulation (PPOS) using machine learning algorithms.

**Methods:**

A retrospective analysis was conducted on the clinical data of 4,216 POR cycles who underwent in vitro fertilization (IVF) / intracytoplasmic sperm injection (ICSI) at Sichuan Jinxin Xinan Women and Children’s Hospital from January 2015 to December 2021. Based on the presence of high-quality cleavage embryos 72 h post-fertilization, the samples were divided into the high-quality cleavage embryo group (*N* = 1950) and the non-high-quality cleavage embryo group (*N* = 2266). Additionally, based on whether high-quality blastocysts were observed following full blastocyst culture, the samples were categorized into the high-quality blastocyst group (*N* = 124) and the non-high-quality blastocyst group (*N* = 1800). The factors influencing the formation of high-quality embryos were analyzed using logistic regression. The predictive models based on machine learning methods were constructed and evaluated accordingly.

**Results:**

Differential analysis revealed that there are statistically significant differences in 14 factors between high-quality and non-high-quality cleavage embryos. Logistic regression analysis identified 14 factors as influential in forming high-quality cleavage embryos. In models excluding three variables (retrieved oocytes, MII oocytes, and 2PN fertilized oocytes), the XGBoost model performed slightly better (AUC = 0.672, 95% CI = 0.636–0.708). Conversely, in models including these three variables, the Random Forest model exhibited the best performance (AUC = 0.788, 95% CI = 0.759–0.818). In the analysis of high-quality blastocysts, significant differences were found in 17 factors. Logistic regression analysis indicated that 13 factors influence the formation of high-quality blastocysts. Including these variables in the predictive model, the XGBoost model showed the highest performance (AUC = 0.813, 95% CI = 0.741–0.884).

**Conclusion:**

We developed a predictive model for the formation of high-quality embryos using machine learning methods for patients with POR undergoing treatment with the PPOS protocol. This model can help infertility patients better understand the likelihood of forming high-quality embryos following treatment and help clinicians better understand and predict treatment outcomes, thus facilitating more targeted and effective interventions.

## Introduction

In vitro fertilization and embryo transfer (IVF-ET), as the primary method for treating infertility, offers hope to the majority of infertile couples to conceive their own children. With the advancement of assisted reproductive technology, the success rate of IVF-ET treatments has significantly improved. Research indicated that transferring high-quality embryos can enhance clinical pregnancy and live birth rates [1–5], whereas low-quality embryos may increase the risk of miscarriage [6]. Embryo quality also had a significant impact on the implantation success rates of natural cycle in vitro fertilization (NC-IVF) [5]. Furthermore, the transplantation of non-optimal embryos was associated with a higher incidence of ectopic pregnancy [7]. Therefore, transferring high-quality embryos can improve clinical pregnancy outcomes.

In the process of ovarian stimulation during IVF-ET, poor ovarian response (POR) is a primary cause for adverse pregnancy outcomes. POR is characterized by high cycle cancellation rates, increased gonadotropin (Gn) dosage, fewer eggs retrieved, suboptimal oocyte quality, and lower clinical pregnancy rates. According to the Poseidon criteria, based on age, antral follicle count (AFC), and anti-Müllerian hormone (AMH), patients are divided into two main categories: ‘unexpected’ poor responders (groups 1 and 2) and ‘expected’ poor responders (groups 3 and 4). POR limits the success of treatment with assisted reproductive technologies (ART) [8], especially for patients in POSEIDON Groups 3 and 4, these patients face a high risk of not producing high-quality embryos suitable for transfer, often leading to multiple ovarian stimulation attempts. This not only increases the physical and emotional strain but also escalates the financial burden [9]. The Progestin Primed Ovarian Stimulation (PPOS) regimen has been clinically confirmed to effectively suppress the luteinizing hormone (LH) peak, with no adverse effect on the quality of eggs and embryos, making it a safe and effective protocol [10–12]. Thus, as controlled ovarian hyperstimulation(COH) trends towards simplification and individualization, the PPOS protocol has gained widespread clinical application.

Machine learning (ML), a subset of artificial intelligence technologies, employs algorithms that adapt and enhance performance by continuously processing tasks and accumulating experience, thereby adjusting parameters automatically without explicit programming [13]. ML encompasses various methodologies, including Logistic Regression (LR), Support Vector Machines(SVM), Decision Trees, Random Forests(RF), Neural Networks(NN), and Naive Bayesian learning. Some studies have confirmed that machine learning approaches can achieve better predictive performance than traditional statistical methods [14, 15]. As ML technology evolves, it promises to enhance IVF success rates by aiding clinical decision-making and predicting reproductive outcomes [16, 17].

However, research remains limited on the factors influencing the formation of high-quality embryos in POSEIDON ‘expected’ patients undergoing the PPOS protocol. This study aims to explore these factors and utilize machine learning techniques to establish a predictive model for ovulation induction outcomes based on individual patient characteristics.

## Materials and methods

### Patients

**Fig. 1:**
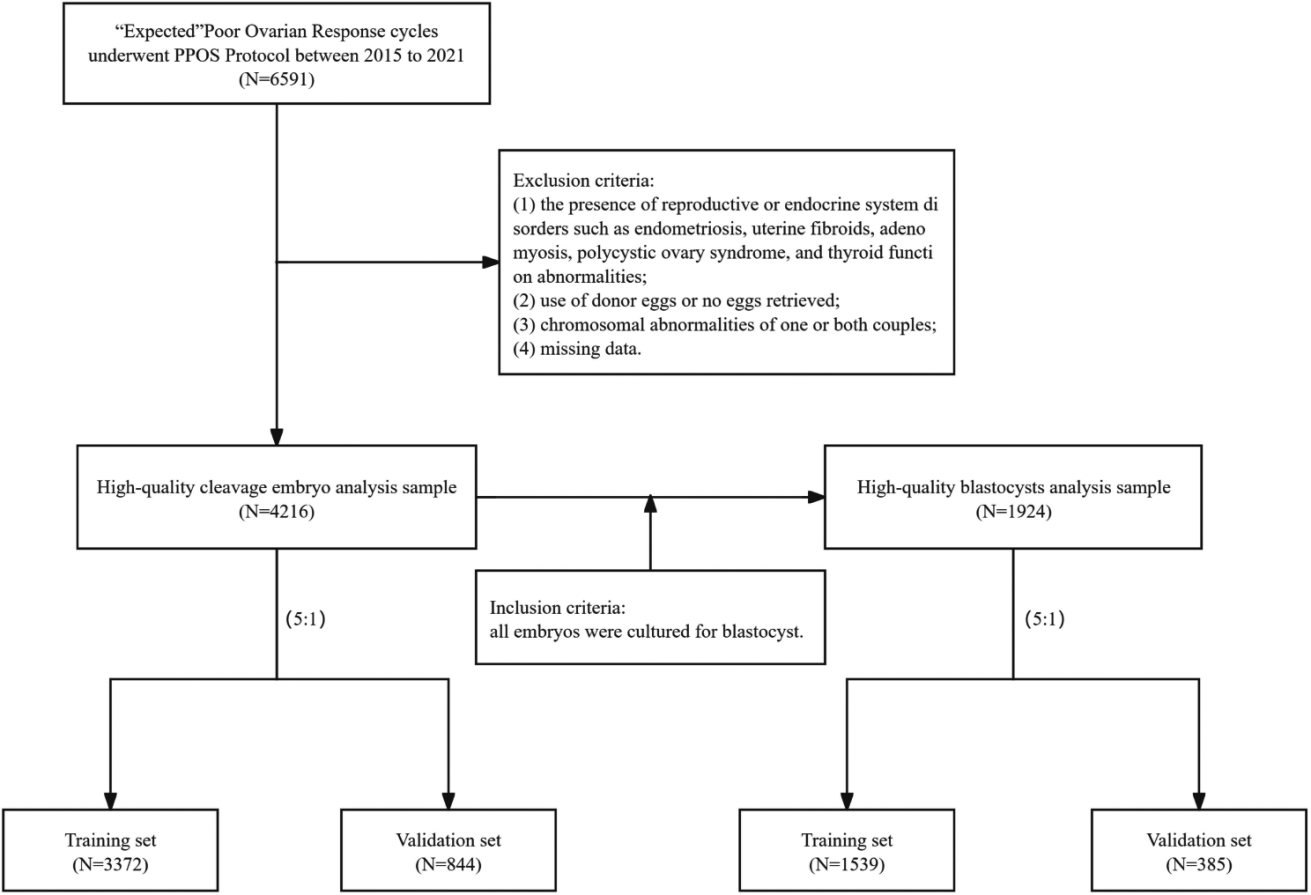
Patient inclusion flowchart

This was a retrospective cohort study conducted at Sichuan Jinxin Xinan Women and Children’s Hospital (China). All the fresh IVF cycles performed in infertile couples from January 2015 to December 2021, were reviewed for possible inclusion. Inclusion criteria: (1) in accordance with the criteria of POSEIDON’s expected low prognosis: AFC < 5 and AMH < 1.2 ng / ml, (2) the ovulation inducing formula was PPOS regimen. Exclusion criteria: (1) the presence of reproductive or endocrine system disorders such as endometriosis, uterine fibroids, adenomyosis, polycystic ovary syndrome, and thyroid function abnormalities; (2) use of donor eggs or no eggs retrieved; (3) chromosomal abnormalities of one or both couples; (4) missing data. The discussion concerning high-quality cleavage embryos is confined to 4,216 cycles. The analysis of high-quality blastocysts is solely restricted to 1,924 cycles who had all their embryos cultured to the blastocyst stage (Fig. 1).

### Variables

Gathering clinical information from patients, which includes the duration of infertility, type of cycle (initial or repeated), nature of infertility (primary or secondary), age, body mass index (BMI), basal follicular-stimulating hormone (FSH), basal estradiol (E2) ,basal progesterone (P), basal LH, FSH/LH, AFC, AMH, FSH on human chorionic gonadotropin (HCG) day, LH on HCG day, P on HCG day, E2 on HCG day, number of follicles ≥ 14 mm on HCG day, total dose of gonadotropin (Gn), dosing days of Gn, the number of oocytes retrieved, MII oocytes and 2PN fertilized oocytes. In addition to the aforementioned variables, we have also incorporated delta FSH, delta LH, delta P, and delta E2, which represent the changes in hormonal levels from the initiation day to the day of HCG day. In the analysis of high-quality blastocysts, we also collected data on the number of cleavage embryos and high-quality cleavage embryos.

### Clinical protocol

Patients [18, 19] received oral administration of medroxyprogesterone acetate (MPA, Xianju Pharmaceutical, Zhejiang, China) at a dosage of 6–10 mg/day from the 2nd to the 5th days of the menstrual cycle. On the same days, FSH (Urofollitropin, Lizhu Pharmaceutical Group, Shanghai, China) was injected at a dosage of 150–300 IU per day. During the ovarian stimulation period, follicle development and serum levels of LH and E2 were monitored to adjust the dose of Gn. When at least one follicles reached a diameter of ≥ 17 mm, an injection of HCG (Merck Serono, Switzerland or Lizhu Pharmaceutical, China) and/or a GnRH agonist (Ferring Pharmaceutical, Switzerland) was used as the trigger. Oocyte retrieval was then performed 34–36 h later.

### Embryo quality assessment

For cleavage embryos, embryos were assigned a subjective score based on the regularity or symmetry of blastomere size, the quality of the cytoplasm, and the degree of embryonic fragmentation in accordance with the specifications of Cummins et al [20]. Thus, a badly fragmented, irregularly cleaved embryo with a patchy or grainy cytoplasm would be assessed as grade IV, whereas an embryo of the highest quality would be assessed as grade I. In the present study, grade I and II embryos were defined as high quality; grade III and IV were defined as low quality. For blastocyst evaluation, we use the Gardner [21] criteria to grade blastocysts into six categories based on the size of the blastocoel, the development of the inner cell mass, and the trophectoderm. Grades 1–3 represent lower quality, while grades 4–6 indicate higher quality.

### Statistical analysis

All statistical analyses and model building were conducted using R software (version 4.3.1), utilizing packages such as randomForest, nnet, xgboost, e1071, rpart, caret, pROC, and ggplot2. The data set of the patients was randomly divided into the training set and the validation set (5:1). Descriptive statistics of quantitative and qualitative data were presented as mean (SD) and numbers (percentages), respectively. Based on the data, the T-test was conducted for normally distributed continuous variables, the Mann–Whitney U test was conducted for non-normally distributed continuous variables, and the Chi-squared test was conducted for classified variables. Univariate and multivariate logistic regression analysis was conducted to identify factors influencing the formation of high-quality embryos in patients. Statistical significance was considered at *P* < 0.05. Subsequently, predictive models were constructed using LR, RF, NN, XGBoost, SVM, NB, Decision Trees, and K-Nearest Neighbors (KNN). Model training involved the use of ten-fold cross-validation and grid search to determine the optimal parameters for each algorithm, aiming to enhance model performance. The performance of each model was evaluated using the area under the receiver operating characteristic curve.

## Results

### High-quality cleavage embryos

#### Analysis of baseline information

From January 2015 to December 2021, 4,216 cycles who used the PPOS protocol and met the POSEIDON criteria for the expected POR group were included in this study. These cycles were allocated into either training set (*N* = 3372) or validation set (*N* = 844) for model establishment and validation. The baseline characteristics were shown in Table 1. In the training set, 1555 cycles (46.1%) achieved high-quality cleavage embryos. There were no statistically significant differences in the cycle type, infertility type, duration of infertility, age, BMI, basal FSH, basal LH, FSH/LH, basal P, basal E2, AFC, AMH, and the formation of high-quality cleavage embryos between the training set and the validation set (*P* > 0.05).

**Table 1:** Baseline characteristics of study population

Characteristics	Validation set (*N* = 844)	Training set (*N* = 3372)	*P* value
Cycle Type			0.780
First Cycle	352 (41.7%)	1386 (41.1%)	
Repeat Cycle	492 (58.3%)	1986 (58.9%)	
Type of infertility			0.782
Primary infertility	265 (31.4%)	1078 (32.0%)	
Secondary infertility	579 (68.6%)	2294 (68.0%)	
Duration of infertility (years)	5.29 ± 4.63	5.23 ± 4.62	0.721
Age (years)	37.8 ± 5.57	37.5 ± 5.59	0.153
BMI	22.4 ± 2.79	22.3 ± 2.87	0.159
Basal FSH (mIU/mL)	13.1 ± 7.58	12.8 ± 7.38	0.265
Basal LH (mIU/mL)	5.17 ± 4.64	5.01 ± 4.48	0.361
FSH/LH	3.07 ± 1.67	3.29 ± 8.31	0.165
Basal P (ng/mL)	0.91 ± 2.03	0.92 ± 1.75	0.932
Basal E2 (pg/mL)	71.2 ± 134	69.3 ± 84.5	0.698
AFC	2.72 ± 1.03	2.75 ± 1.03	0.484
AMH (ng/mL)	0.49 ± 0.30	0.51 ± 0.31	0.368
High-quality cleavage embryo formation			0.750
No	449 (53.2%)	1817 (53.9%)	
Yes	395 (46.8%)	1555 (46.1%)	

Continuous variables are expressed as mean ± SD, categorical variables as absolute frequencies, n (%)

BMI, body mass index; FSH, follicular-stimulating hormone; LH, luteinizing hormone; P, progesterone; E2, estradiol; AFC, antral follicle count; AMH, anti-Müllerian hormone

#### Differences in fourteen factors between high-quality and non-high-quality cleavage embryo groups

The participants were divided into two groups based on the acquisition of high-quality cleavage embryos: the high-quality cleavage embryo group (*N* = 1950) and the non-high-quality cleavage embryo group (*N* = 2266). In the high-quality cleavage embryo group, the AFC, AMH, P on HCG day, E2 on HCG day, delta E2, and the number of follicles ≥ 14 mm on HCG day were significantly higher compared to the non-high-quality embryo group (*P* < 0.05). Women who obtained high-quality cleavage embryos used more Gn for longer durations and retrieved more oocytes, M II oocytes, and 2PN fertilized oocytes (*P* < 0.05). These findings suggest that patients in the high-quality cleavage embryo group exhibit higher ovarian responsiveness and better ovarian reserve (Table 2).

**Table 2:** Comparison between the high-quality cleavage embryo group and the non-high-quality cleavage embryo group

Characteristics	Non-high-quality cleavage embryo group (*N* = 2266)	High-quality cleavage embryo group (*N* = 1950)	*P* value
Cycle Type			0.784
First Cycle	939(41.44%)	799(40.97%)	
Repeat Cycle	1327(58.56%)	1151(59.03%)	
Type of infertility			0.331
Primary infertility	737(32.52%)	606(31.08%)	
Secondary infertility	1529(67.48%)	1344(68.92%)	
Duration of infertility (years)	5.30 ± 4.57	5.16 ± 4.69	**0.043**
Age (years)	37.62 ± 5.61	37.44 ± 5.56	0.278
BMI	22.27 ± 2.85	22.36 ± 2.86	0.192
Basal FSH (mIU/mL)	13.10 ± 7.78	12.62 ± 6.98	0.140
Basal LH (mIU/mL)	5.27 ± 5.30	4.78 ± 3.35	0.157
FSH/LH	3.21 ± 7.23	3.28 ± 7.74	0.563
Basal P (ng/mL)	0.93 ± 1.88	0.91 ± 1.74	0.300
Basal E2 (pg/mL)	69.88 ± 79.84	69.53 ± 112.95	0.182
AFC	2.71 ± 1.03	2.78 ± 1.02	**0.020**
AMH (ng/mL)	0.48 ± 0.30	0.54 ± 0.31	**7.18E-10**
FSH on HCG day (mIU/mL)	19.21 ± 6.16	19.12 ± 5.77	0.909
LH on HCG day (mIU/mL)	3.43 ± 2.75	2.84 ± 2.38	**4.71E-13**
P on HCG day (ng/mL)	0.76 ± 0.60	0.80 ± 0.82	**0.047**
E2 on HCG day (pg/mL)	936.56 ± 605.29	1171.06 ± 772.61	**1.02E-29**
Delta FSH	9.76 ± 6.73	9.83 ± 6.48	0.497
Delta LH	-0.91 ± 3.12	-1.33 ± 2.77	**2.37E-06**
Delta P	0.04 ± 0.87	0.06 ± 1.64	0.135
Delta E2	862.70 ± 613.65	1101.97 ± 774.65	**1.85E-31**
Number of follicles ≥ 14 mm on HCG day	2.25 ± 1.42	2.96 ± 1.85	**1.09E-46**
Total dose of Gn (IU)	1701.17 ± 832.45	1825.58 ± 764.52	**1.59E-10**
Dosing days of Gn (day)	8.31 ± 3.09	8.74 ± 2.98	**4.90E-08**
Retrieved oocytes	1.97 ± 1.35	2.93 ± 2.00	**1.46E-80**
MII oocytes	1.73 ± 1.23	2.73 ± 1.84	**1.03E-100**
2PN fertilized oocytes	1.07 ± 1.04	2.24 ± 1.50	**3.79E-204**

Continuous variables are expressed as mean ± SD, categorical variables as absolute frequencies, n (%)

BMI, body mass index; FSH, follicular-stimulating hormone; LH, luteinizing hormone; P, progesterone; E2, estradiol; AFC, antral follicle count; AMH, anti-Müllerian hormone; Gn, gonadotropin; HCG, human chorionic gonadotropin

#### Fourteen factors are associated with the formation of high-quality cleavage embryos

The overall rate of obtaining high-quality cleavage embryos was 46.3%. In the univariate logistic regression analysis, fourteen factors were associated with the formation of high-quality cleavage embryos: basal FSH, basal LH, AFC, AMH, LH on HCG day, E2 on HCG day, delta LH, delta E2, number of follicles ≥ 14 mm on HCG day, total dose of Gn, dosing days of Gn, number of retrieved oocytes, MII oocytes, and 2PN fertilized oocytes (*P* < 0.05). After adjusting for confounding factors, basal LH, delta LH, number of retrieved oocytes, and 2PN fertilized oocytes were identified as independent predictors of obtaining high-quality cleavage embryos (*P* < 0.05) (Table 3).

**Table 3:** Univariate and multivariate logistic regression analysis

Characteristics	Univariate		Multivariate	
	OR (95%CI)	*P* value	OR (95%CI)	*P* value
Cycle Type	1.019(0.901–1.153)	0.760		
First Cycle				
Repeat Cycle				
Type of infertility	1.069(0.939–1.218)	0.315		
Primary infertility				
Secondary infertility				
Duration of infertility (years)	0.993(0.981–1.007)	0.327		
Age (years)	0.994(0.983–1.005)	0.278		
BMI	1.011(0.990–1.033)	0.303		
Basal FSH (mIU/mL)	0.991(0.983–0.999)	**0.037**	1.010(0.999–1.021)	0.073
Basal LH (mIU/mL)	0.974(0.959–0.989)	**0.001**	0.972(0.952–0.992)	**0.006**
FSH/LH	1.001(0.993–1.009)	0.766		
Basal P (ng/mL)	0.995(0.963–1.029)	0.785		
Basal E2 (pg/mL)	1.000(0.999–1.001)	0.907		
AFC	1.073(1.012–1.138)	**0.019**	0.952(0.889–1.020)	0.160
AMH (ng/mL)	1.923(1.574–2.350)	**1.630E-10**	1.201(0.940–1.534)	0.143
FSH on HCG day (mIU/mL)	0.998(0.987–1.008)	0.631		
LH on HCG day (mIU/mL)	0.908(0.885–0.932)	**8.275E-13**	0.986(0.949–1.025)	0.479
P on HCG day (ng/mL)	1.082(0.986–1.186)	0.096		
E2 on HCG day (pg/mL)	1.001(1.000-1.001)	**7.035E-26**	1.000(0.999–1.001)	0.730
Delta FSH	1.002(0.993–1.011)	0.727		
Delta LH	0.952(0.933–0.973)	**5.284E-06**	0.969(0.940-1.000)	**0.048**
Delta P	1.011(0.963–1.061)	0.656		
Delta E2	1.001(1.000-1.001)	**1.753E-26**	1.000(0.999–1.001)	0.608
Number of follicles ≥ 14 mm on HCG day	1.321(1.268–1.376)	**4.804E-41**	0.967(0.890–1.050)	0.422
Total dose of Gn (IU)	1.000(1.000–1.000)	**6.165E-07**	1.000(1.000–1.000)	0.400
Dosing days of Gn (day)	1.048(1.026–1.069)	**8.04489E-06**	1.016(0.974–1.059)	0.460
Retrieved oocytes	1.442(1.381–1.505)	**1.716E-62**	0.805(0.701–0.925)	**0.002**
MII oocytes	1.591(1.515–1.671)	**2.515E-77**	1.001(0.855–1.173)	0.986
2PN fertilized oocytes	2.372(2.213–2.542)	**3.198E-132**	3.086(2.759–3.452)	**1.202E-86**

BMI, body mass index; FSH, follicular-stimulating hormone; LH, luteinizing hormone; P, progesterone; E2, estradiol; AFC, antral follicle count; AMH, anti-Müllerian hormone; Gn, gonadotropin; HCG, human chorionic gonadotropin

#### Construction and evaluation of the prediction model

**Fig. 2:**
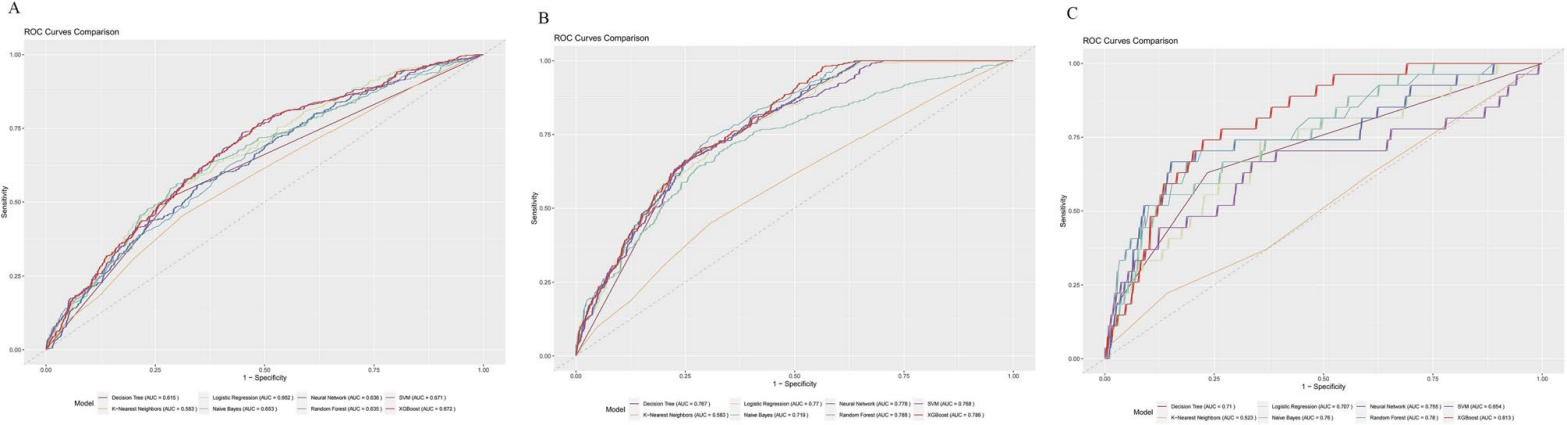
(**A**) ROC curve of the M1; (**B**) ROC curve of the M2; (**C**) ROC curve of the M3

The factors significantly associated with the formation of high-quality cleavage embryos were selected for the construction of a predictive model. In the initial phase of constructing our predictive model, we did not include the number of retrieved oocytes, M II oocytes, and 2PN fertilized oocytes (Table 7). The performance evaluation and ROC curves of different models are available in Table 8; Fig. 2. In M1, the AUC values for all models were not very satisfactory, with the XGBoost model performing slightly better than others (AUC = 0.672, 95% CI = 0.636–0.708). In M2, although the performances of the models were comparable, the RF model exhibited superior performance (AUC = 0.788, 95% CI = 0.759–0.818). Additionally, in both M1 and M2, the performance of the KNN model was significantly below the expected standards.

### High-quality blastocysts

#### Analysis of baseline information

Cycles who underwent culture of all embryos to the blastocyst stage were included in the study (*N* = 1924). These cycles were divided into a training set (*N* = 1539) or a validation set (*N* = 385) for the development and validation of the model. Baseline characteristics are shown in Table 4. In the training set, 97 cycles (6.3%) obtained high-quality blastocysts. There were no statistically significant differences between the training and validation sets in terms of cycle type, infertility type, duration of infertility, age, BMI, baseline FSH, baseline LH, FSH/LH , basal P, basal E2, AFC, AMH, and the formation of high-quality blastocysts (*P* > 0.05).

**Table 4:** Baseline characteristics of study population

Characteristics	Validation set (*N* = 385)	Training set (*N* = 1539)	*P* value
Cycle Type			0.308
First Cycle	147 (38.2%)	634 (41.2%)	
Repeat Cycle	238 (61.8%)	905 (58.8%)	
Type of infertility			0.434
Primary infertility	132 (34.3%)	493 (32.0%)	
Secondary infertility	253 (65.7%)	1046 (68.0%)	
Duration of infertility (years)	5.10 ± 4.18	5.30 ± 4.60	0.399
Age (years)	37.9 ± 5.59	37.7 ± 5.55	0.560
BMI	22.1 ± 2.82	22.3 ± 2.86	0.362
Basal FSH (mIU/mL)	13.5 ± 8.78	13.3 ± 7.77	0.560
Basal LH (mIU/mL)	5.43 ± 5.59	5.30 ± 5.14	0.677
FSH/LH	3.07 ± 1.68	3.27 ± 8.67	0.386
Basal P (ng/mL)	0.80 ± 1.29	0.95 ± 2.01	0.082
Basal E2 (pg/mL)	72.3 ± 91.5	71.0 ± 82.3	0.796
AFC	2.71 ± 1.03	2.70 ± 1.03	0.944
AMH (ng/mL)	0.47 ± 0.29	0.47 ± 0.29	0.677
High-quality blastocysts formation			0.695
No	358 (93.0%)	1442 (93.7%)	
Yes	27 (7.01%)	97 (6.30%)	

Continuous variables are expressed as mean ± SD, categorical variables as absolute frequencies, n (%)

BMI, body mass index; FSH, follicular-stimulating hormone; LH, luteinizing hormone; P, progesterone; E2, estradiol; AFC, antral follicle count; AMH, anti-Müllerian hormone

#### Differences in seventeen factors between high-quality and non-high-quality blastocyst groups

The participants were divided into two groups based on the acquisition of high-quality blastocyst: the high-quality blastocyst group (*N* = 124) and the non-high-quality blastocyst group (*N* = 1800). In the high-quality blastocyst group, basal P, AFC, AMH, FSH on HCG day, P on HCG day, E2 on HCG day, delta FSH, delta E2, number of follicles ≥ 14 mm on HCG day, retrieved oocytes, MII oocytes, 2PN fertilized oocytes, cleavage embryos, and high-quality cleavage embryos were all significantly higher compared to the non-high-quality blastocyst group. Conversely, age, BMI, and LH on HCG day were significantly lower in the high-quality blastocyst group (*P* < 0.05) (Table 5).

**Table 5:** Comparison between the high-quality blastocyst group and the non-high-quality blastocyst group

Characteristics	Non-high-quality blastocyst group (*N* = 1800)	High-quality blastocyst group (*N* = 124)	*P* value
Cycle Type			0.875
First Cycle	732(40.67%)	49(39.52%)	
Repeat Cycle	1068(59.33%)	75(60.48%)	
Type of infertility			0.301
Primary infertility	579(32.17%)	46(37.10%)	
Secondary infertility	1221(67.83%)	78(62.90%)	
Duration of infertility (years)	5.28 ± 4.51	5.06 ± 4.56	0.317
Age (years)	37.87 ± 5.57	35.39 ± 4.87	**6.51E-07**
BMI	22.31 ± 2.87	21.54 ± 2.45	**0.020**
Basal FSH (mIU/mL)	13.35 ± 8.02	12.83 ± 7.47	0.360
Basal LH (mIU/mL)	5.28 ± 4.97	6.06 ± 8.14	0.508
FSH/LH	3.25 ± 8.03	2.93 ± 2.10	0.175
Basal P (ng/mL)	0.89 ± 1.82	1.32 ± 2.69	**0.017**
Basal E2 (pg/mL)	70.47 ± 80.01	82.46 ± 130.57	0.433
AFC	2.68 ± 1.03	3.03 ± 0.95	**0.0002**
AMH (ng/mL)	0.47 ± 0.30	0.52 ± 0.28	**0.037**
FSH on HCG day (mIU/mL)	19.19 ± 6.26	20.13 ± 5.47	**0.020**
LH on HCG day (mIU/mL)	3.54 ± 2.76	3.11 ± 2.86	**0.009**
P on HCG day (ng/mL)	0.77 ± 0.65	0.80 ± 0.44	**0.036**
E2 on HCG day (pg/mL)	897.46 ± 593.19	1148.06 ± 751.30	**3.36E-06**
Delta FSH	9.66 ± 6.81	10.99 ± 6.55	**0.006**
Delta LH	-0.86 ± 3.17	-1.02 ± 3.39	0.180
Delta P	0.05 ± 0.87	0.07 ± 0.46	0.558
Delta E2	820.94 ± 599.70	1082.54 ± 759.60	**1.97E-06**
Number of follicles ≥ 14 mm on HCG day	2.14 ± 1.41	2.72 ± 1.51	**7.48E-07**
Total dose of Gn (IU)	1686.66 ± 843.79	1740.12 ± 698.21	0.136
Dosing days of Gn (day)	8.27 ± 3.16	8.41 ± 2.57	0.148
Retrieved oocytes	1.82 ± 1.27	2.47 ± 1.68	**8.34E-07**
MII oocytes	1.59 ± 1.19	2.31 ± 1.56	**2.56E-09**
2PN fertilized oocytes	0.90 ± 1.03	1.44 ± 1.28	**3.42E-07**
Cleavage embryo	1.07 ± 1.06	2.00 ± 1.21	**1.40E-19**
High-quality cleavage embryo	0.03 ± 0.36	0.27 ± 0.64	**7.72E-25**

Continuous variables are expressed as mean ± SD, categorical variables as absolute frequencies, n (%)

BMI, body mass index; FSH, follicular-stimulating hormone; LH, luteinizing hormone; P, progesterone; E2, estradiol; AFC, antral follicle count; AMH, anti-Müllerian hormone; Gn, gonadotropin; HCG, human chorionic gonadotropin

#### Thirteen factors are associated with the formation of high-quality blastocysts

The overall rate of obtaining high-quality blastocysts was 6.40%. AFC, E2 on HCG day, delta E2, number of follicles ≥ 14 mm on HCG day, retrieved oocytes, MII oocytes, and 2PN fertilized oocytes are not only influencing factors for high-quality cleavage embryos but also for high-quality blastocysts (*P* < 0.05). In addition, age, BMI, basal P, delta FSH, number of cleavage embryos, and high-quality cleavage embryos are also influencing factors for high-quality blastocysts (*P* < 0.05). After adjusting for confounding factors, age, AFC, number of 2PN fertilized oocytes, cleavage embryos, and high-quality cleavage embryos were identified as independent predictors for the formation of high-quality blastocysts (*P* < 0.05) (Table 6).

**Table 6:** Univariate and multivariate logistic regression analysis

Characteristics	Univariate		Multivariate	
	OR (95%CI)	*P* value	OR (95%CI)	*P* value
Cycle Type	1.049(0.723–1.522)	0.801		
First Cycle				
Repeat Cycle				
Type of infertility	0.804(0.551–1.173)	0.258		
Primary infertility				
Secondary infertility				
Duration of infertility (years)	0.989(0.949–1.031)	0.599		
Age (years)	0.924(0.894–0.955)	**2.02E-06**	0.918(0.884–0.954)	**9.178E-06**
BMI	0.903(0.843–0.967)	**0.004**	0.936(0.869–1.008)	0.080
Basal FSH (mIU/mL)	0.991(0.967–1.016)	0.489		
Basal LH (mIU/mL)	1.021(0.995–1.048)	0.115		
FSH/LH	0.951(0.844–1.071)	0.404		
Basal P (ng/mL)	1.073(1.010–1.140)	**0.023**	1.052(0.985–1.124)	0.129
Basal E2 (pg/mL)	1.001(1.000-1.003)	0.131		
AFC	1.426(1.178–1.727)	**0.0003**	1.396(1.132–1.721)	**0.002**
AMH (ng/mL)	1.789(0.978–3.270)	0.059		
FSH on HCG day (mIU/mL)	1.023(0.996–1.051)	0.102		
LH on HCG day (mIU/mL)	0.937(0.867–1.011)	0.095		
P on HCG day (ng/mL)	1.071(0.836–1.372)	0.588		
E2 on HCG day (pg/mL)	1.000(1.000-1.001)	**1.799E-05**	0.999(0.995–1.002)	0.500
Delta FSH	1.027(1.002–1.053)	**0.035**	1.021(0.992–1.051)	0.165
Delta LH	0.984(0.928–1.042)	0.577		
Delta P	1.022(0.817–1.277)	0.851		
Delta E2	1.000(1.000-1.001)	**9.871E-06**	1.001(0.998–1.005)	0.420
Number of follicles ≥ 14 mm on HCG day	1.238(1.119–1.369)	**3.37E-05**	0.906(0.727–1.129)	0.380
Total dose of Gn	1.000(1.000–1.000)	0.490		
Dosing days of Gn	1.014(0.958–1.073)	0.632		
Retrieved oocytes	1.296(1.168–1.439)	**1.115E-06**	0.659(0.424–1.023)	0.063
MII oocytes	1.378(1.233–1.541)	**1.781E-08**	0.98(0.581–1.653)	0.940
2PN fertilized oocytes	1.393(1.218–1.593)	**1.223E-06**	0.665(0.509–0.868)	**0.003**
Cleavage embryo	1.705(1.486–1.957)	**2.8E-14**	3.244(2.234–4.709)	**6.17E-10**
High-quality cleavage embryo	2.070(1.449–2.957)	**6.344E-05**	1.574(1.030–2.406)	**0.036**

BMI, body mass index; FSH, follicular-stimulating hormone; LH, luteinizing hormone; P, progesterone; E2, estradiol; AFC, antral follicle count; AMH, anti-Müllerian hormone; Gn, gonadotropin; HCG, human chorionic gonadotropin

#### Construction and evaluation of the prediction model

Factors significantly associated with the formation of high-quality blastocysts were used to construct the models (Table 7). The performance of each model and the ROC curves can be seen in Table 8; Fig. 2. Similar to the models predicting high-quality cleavage embryos, the KNN model performed far below the acceptable range. Among the other models, XGBoost achieved the best performance (AUC = 0.813, 95% CI = 0.741–0.884).

**Table 7:** Factors included in model construction

Characteristics	Model 1	Model 2	Model 3
Age (years)			Y
BMI			Y
Basal FSH (mIU/mL)	Y	Y	
Basal LH (mIU/mL)	Y	Y	
Basal P (ng/mL)			Y
AFC	Y	Y	Y
AMH (ng/mL)	Y	Y	
LH on HCG day (mIU/mL)	Y	Y	
E2 on HCG day (pg/mL)	Y	Y	Y
Delta FSH			Y
Delta LH	Y	Y	
Delta E2	Y	Y	Y
Number of follicles ≥ 14 mm on HCG day	Y	Y	Y
Total dose of Gn (IU)	Y	Y	
Dosing days of Gn (days)	Y	Y	
Retrieved oocytes		Y	Y
MII oocytes		Y	Y
2PN fertilized oocytes		Y	Y
Cleavage embryo			Y
High-quality cleavage embryo			Y

M1 and M2 for predicting high-quality cleavage embryos, M3 for predicting high-quality blastocysts

BMI, body mass index; FSH, follicular-stimulating hormone; LH, luteinizing hormone; P, progesterone; E2, estradiol; AFC, antral follicle count; AMH, anti-Müllerian hormone; Gn, gonadotropin; HCG, human chorionic gonadotropin

**Table 8:** Performance of three predictive models

	M1	M2	M3
Logistic Regression	0.662 (0.626–0.698)	0.770 (0.739–0.801)	0.707 (0.600-0.814)
Random Forest	0.635 (0.598–0.672)	**0.788 (0.759–0.818)**	0.780 (0.682–0.878)
Neural Network	0.636 (0.599–0.673)	0.778 (0.747–0.808)	0.755 (0.645–0.864)
Support Vector Machine	0.671 (0.635–0.708)	0.768 (0.737–0.799)	0.654 (0.520–0.787)
Naive Bayes	0.653 (0.616–0.690)	0.719 (0.684–0.754)	0.760 (0.668–0.852)
XGBoost	**0.672 (0.636–0.708)**	0.786 (0.756–0.816)	**0.813 (0.741–0.884)**
Decision Tree	0.615 (0.582–0.647)	0.767 (0.738–0.795)	0.710 (0.610–0.810)
K-Nearest Neighbors	0.583 (0.545–0.621)	0.583 (0.545–0.622)	0.523 (0.407–0.639)

M1(Predicting high-quality cleavage embryos): basal FSH, basal LH, AFC, AMH, LH on HCG day, E2 on HCG day, delta LH, delta E2, number of follicles ≥ 14 mm on HCG day, total dose of Gn, Dosing days of Gn

M2(Predicting high-quality cleavage embryos): basal FSH, basal LH, AFC, AMH, LH on HCG day, E2 on HCG day, delta LH, delta E2, number of follicles ≥ 14 mm on HCG day, total dose of Gn, Dosing days of Gn, retrieved oocytes, MII oocytes, 2PN fertilized oocytes

M3(predicting high-quality blastocysts): age, BMI, basal P, AFC, E2 on HCG day, delta FSH, delta E2, number of follicles ≥ 14 mm on HCG day, retrieved oocytes, MII oocytes, 2PN fertilized oocytes, cleavage embryo, high-quality cleavage embryo

## Discussion

Selecting high-quality embryos is crucial for successful pregnancy. In our study, we established three predictive models: M1 and M2 are designed to predict the formation of high-quality cleavage embryos, M3 is aimed at predicting the formation of high-quality blastocysts. The performance of these models demonstrates that they have predictive value for the formation of high-quality cleavage embryos or blastocysts. However, all models in M1 performed poorly, with AUC values below acceptable levels. This could be attributed to the composition of our cohort or possibly the factors included. By incorporating three additional variables—retrieved oocytes, MII oocytes, and 2PN fertilized oocytes—into M2, the model’s performance significantly improved, with a notable increase in AUC. This suggests that these variables play a crucial predictive role in the formation of high-quality cleavage embryos.

Our research has found that indicators such as age, AMH, AFC, FSH, and LH are associated with the formation of high-quality embryos. Previous research [22] has revealed that with the increase in women’s age, the incidence of aneuploidy in embryos and oocytes, as well as the decline in embryo quality, increase. These changes result in a reduced number of viable embryos and an increased risk of miscarriage. Some studies [22–33] indicated that as women age and baseline ovarian markers change, such as lower AMH and AFC, along with higher basal FSH, the prognosis was observed to worsen. High E2 levels are common during COH, and elevated E2 may affect embryo quality and further affect pregnancy outcomes in IVF [34–37]. The results of a study [34] showed that a decline of more than 30% in donor serum E2 levels during the ovarian stimulation process adversely affected the quality of recipient embryos. A decrease in E2 levels adversely impacts embryo quality, leading to reduced clinical pregnancy rates, ongoing pregnancy rates, and an increased rate of early miscarriage [38]. Our study aligns with previous findings, observing that for both high-quality cleavage embryos and blastocysts, the parameters of AFC, AMH, E2 on HCG day, and delta E2 are significantly higher in high-quality embryos compared to non-high-quality embryos. While age shows no significant difference between the high-quality and non-high-quality cleavage embryo groups, there is a notable difference in the blastocyst groups. This suggests that for older patients with expected POR, the decision to culture all embryos to the blastocyst stage should be made with caution.

The two-cell theory suggested that normal follicular growth and maturation require both LH and FSH, and that the levels and ratios of these hormones are critical at different points in the menstrual cycle [39]. The fluctuations in LH levels during the follicular phase significantly impact the morphological and functional changes of the oocytes, thereby affecting their meiotic state and the fertilization capability of the zygote [40]. A prospective study [41] indicated that a decrease in LH levels during controlled ovarian stimulation was associated with a decline in oocyte and embryo quality. Previous studies on long and antagonist protocols [42, 43] indicated that LH levels below 0.5 IU/L or 1.0 IU/L on the day of triggering are associated with reduced oocyte retrieval rates and fewer high-quality embryos. In contrast, our study shows that the average LH levels in all four groups of patients were significantly higher than these thresholds, and our protocol was the PPOS protocol. This might explain the different outcomes observed in our study. Additionally, research had demonstrated that basal FSH levels are correlated with overall ovarian responsiveness [44]. Our study results showed that although there was no significant statistical difference in basal FSH levels between the high-quality cleavage/blastocyst group and the non-high-quality group, basal FSH was significantly associated with the formation of high-quality cleavage embryos in the univariate logistic regression analysis. This discrepancy may be due to our target population consisting of expected POR rather than unexpected POR patients.

Ovarian stimulation inducing multi-follicular growth can lead to the collection of multiple oocytes. Several studies had suggested that a higher number of retrieved oocytes was associated with improved outcomes [45–47], whereas contrasting research has posited that an increase in the number of retrieved oocytes was correlated with a decline in oocyte quality, subsequently leading to embryos with reduced developmental potential [48]. Our results indicate that the number of oocytes retrieved in the high-quality cleavage/blastocyst groups was significantly higher than in the non-high-quality groups, suggesting that a greater number of retrieved oocytes increases the likelihood of obtaining high-quality embryos. However, multivariate logistic regression indicates that an increased number of retrieved oocytes is an independent risk factor for high-quality cleavage embryos (OR = 0.805, 95% CI = 0.701–0.925). Therefore, there may be an optimal range of oocyte numbers that can enhance embryo quality and optimize live birth rates. It is well-known that the number of 2PN fertilized oocytes reflects, to a certain extent, the quality of oocytes, laboratory culture conditions, and operational techniques. High-quality embryos originate from a good ovarian response, and the number of 2PN fertilized oocytes can effectively reflect the quality of both sperm and oocytes, significantly influencing the formation of high-quality embryos [49]. Therefore, clinicians should thoroughly evaluate ovarian reserve function before treating patients, administer ovarian stimulation medications, and strive to improve oocyte quality and increase the number of 2PN fertilized oocytes to enhance the rate of high-quality embryo formation.

ML enables the interpretation of data and the construction of prediction models, has been increasingly utilized in clinical settings, particularly within complex systems involving multiple variables [50, 51]. Our study is the first to employ various machine learning methods, utilizing patient clinical characteristics and laboratory data, to establish a predictive model for high-quality embryo formation in expected POR patients undergoing PPOS protocol. Regardless of whether it was M1, M2, or M3, the models built using the KNN method consistently underperformed compared to other machine learning techniques, suggesting that our data might not be suitable for the KNN method. In contrast, XGBoost performed well across Models M1, M2, and M3. XGBoost is a tree-based algorithm that predicts by constructing multiple decision trees. It has natural robustness to outliers, which means that outliers are less likely to significantly impact the choice of split points in the model. This robustness could be a critical factor in its superior performance across various models.

Several limitations of this study warrant attention. First, it is a retrospective study based on data from a single center, there is a certain risk of bias, and the collected data inevitably contain human errors. Second, the study included a limited set of clinical features and did not conduct stratified analyses of factors such as male semen quality, thus presenting certain limitations. Future efforts will aim to expand the range of predictive factors screened to further optimize the model. Additionally, this model was developed based on patients with POR and may not be applicable to other groups. Finally, although the formation of high-quality embryos can reflect embryo quality, it does not fully represent pregnancy outcomes. Future studies could expand the sample size and undertake prospective, multicenter research to provide references for the clinical treatment of infertility.

## Conclusion

In summary, our study identified basal LH, delta LH, the number of retrieved oocytes, and 2PN fertilized oocytes as independent factors influencing high-quality cleavage embryos, while age, AFC, the number of 2PN fertilized oocytes, cleavage embryo, and high-quality cleavage embryo were independent factors for high-quality blastocysts. Additionally, we integrated readily available predictive variables such as E2 on HCG day, delta E2, delta LH, retrieved oocytes, MII oocytes, and 2PN fertilized oocytes to construct predictive models. These models are used to forecast the formation of high-quality cleavage embryos and blastocysts in women with POR undergoing treatment with the PPOS protocol.

## Data Availability

The data used in this article were obtained from the Sichuan Jinxin Xinan Women and Children’s Hospital by request. Upon data request, the corresponding author would obtain permission from Sichuan Jinxin Xinan Women and Children’s Hospital before sharing them.
